# Graphitization of nitrogen-bearing organic-like carbonaceous material in a dust particle from Chang’e-6 lunar samples

**DOI:** 10.1093/nsr/nwag573

**Published:** 2026-09-04

**Authors:** Tian Zhang, Hong Tang, Xiongyao Li, Jialong Hao, Bing Mo, Yuanyun Wen, Futing Yin, Liming Wan, Chuanjiao Zhou, Wen Yu, Haiyan Long, Jianzhong Liu

**Affiliations:** State Key Laboratory of Critical Mineral Research and Exploration, Institute of Geochemistry, Chinese Academy of Sciences, Guiyang 550081, China; Center for Lunar and Planetary Sciences, Institute of Geochemistry, Chinese Academy of Sciences, Guiyang 550081, China; College of Earth and Planetary Sciences, University of Chinese Academy of Sciences, Beijing 100049, China; State Key Laboratory of Critical Mineral Research and Exploration, Institute of Geochemistry, Chinese Academy of Sciences, Guiyang 550081, China; Center for Lunar and Planetary Sciences, Institute of Geochemistry, Chinese Academy of Sciences, Guiyang 550081, China; College of Earth and Planetary Sciences, University of Chinese Academy of Sciences, Beijing 100049, China; State Key Laboratory of Critical Mineral Research and Exploration, Institute of Geochemistry, Chinese Academy of Sciences, Guiyang 550081, China; Center for Lunar and Planetary Sciences, Institute of Geochemistry, Chinese Academy of Sciences, Guiyang 550081, China; College of Earth and Planetary Sciences, University of Chinese Academy of Sciences, Beijing 100049, China; Key Laboratory of the Earth and Planetary Physics, Institute of Geology and Geophysics, Chinese Academy of Sciences, Beijing 100029, China; Center for Lunar and Planetary Sciences, Institute of Geochemistry, Chinese Academy of Sciences, Guiyang 550081, China; Center for Lunar and Planetary Sciences, Institute of Geochemistry, Chinese Academy of Sciences, Guiyang 550081, China; Center for Lunar and Planetary Sciences, Institute of Geochemistry, Chinese Academy of Sciences, Guiyang 550081, China; College of Earth and Planetary Sciences, University of Chinese Academy of Sciences, Beijing 100049, China; Center for Lunar and Planetary Sciences, Institute of Geochemistry, Chinese Academy of Sciences, Guiyang 550081, China; College of Earth and Planetary Sciences, University of Chinese Academy of Sciences, Beijing 100049, China; Center for Lunar and Planetary Sciences, Institute of Geochemistry, Chinese Academy of Sciences, Guiyang 550081, China; Center for Lunar and Planetary Sciences, Institute of Geochemistry, Chinese Academy of Sciences, Guiyang 550081, China; Center for Lunar and Planetary Sciences, Institute of Geochemistry, Chinese Academy of Sciences, Guiyang 550081, China; College of Earth and Planetary Sciences, University of Chinese Academy of Sciences, Beijing 100049, China; State Key Laboratory of Critical Mineral Research and Exploration, Institute of Geochemistry, Chinese Academy of Sciences, Guiyang 550081, China; Center for Lunar and Planetary Sciences, Institute of Geochemistry, Chinese Academy of Sciences, Guiyang 550081, China; College of Earth and Planetary Sciences, University of Chinese Academy of Sciences, Beijing 100049, China

**Keywords:** carbon-rich dust particles, graphitic carbon, organic-like matter, Chang’e-6 lunar regolith

## Abstract

The delivery and evolution of carbonaceous materials to airless planetary bodies remain poorly constrained. This study characterized a porous, aggregated, carbon-rich dust particle (CE6-CDP) from Chang’e-6 lunar samples. CE6-CDP yielded a mean δ^13^C_VPDB_ value of −25.92 ± 3.62‰ and exhibited significant nitrogen enrichment. Nano-infrared spectroscopy further detected N–H, C=N and C–H bonds, indicating that the carbonaceous material within CE6-CDP shows organic-like signatures. Graphitic carbon with tangled and concentric textures within CE6-CDP exhibited a comparable δ^13^C_VPDB_ value (−27.31‰) but contained low nitrogen, suggesting possible graphitization of some organic precursors. The coexistence of organic-like carbonaceous material and graphitic carbon is consistent with dust ejection during an impact involving a larger carbon-rich projectile. This study indicates that extraterrestrial carbon may constitute an important lunar carbon reservoir and contribute to the dark surfaces of Mercury and Vesta. These findings provide new insights into carbon transport and evolution across airless planetary bodies.

## Teaser

Analysis of a carbon‑rich dust particle returned by China’s Chang’e‑6 mission reveals coexistence of nitrogen‑bearing organic‑like matter and graphitic carbon, indicating impact‑delivered carbon may form an important lunar carbon reservoir and contribute to the dark surfaces of other airless bodies.

## INTRODUCTION

Comets, asteroids and cosmic dust have been identified as important carriers of exogenous extraterrestrial carbon to rocky planetary bodies [1–3]. The mass accretion of extraterrestrial debris onto Earth’s upper atmosphere is governed by two components: rare high-mass impactors and the continuous influx of fine interplanetary dust particles (IDPs) [4]. Current estimates suggest that Earth accretes ~110 ± 55 tons of interplanetary dust per day [4]. Under solar and planetary gravity and size-dependent dynamical processes such as Poynting–Robertson drag, dust particles, including those from the Kuiper Belt, can migrate to near-Earth orbits (~1 AU) [5]. The Moon, Earth’s natural satellite, may capture large quantities of dust particles from space.

This lunar dust accretion has been documented by the Lunar Atmosphere and Dust Environment Explorer, which detected a persistent asymmetric dust cloud surrounding the Moon. This dust cloud is generally attributed to hypervelocity impacts of cometary dust on the lunar surface [6]. Dynamical simulations further suggest that the present-day meteoroid mass flux from asteroidal and cometary sources to the Moon reaches several hundred tons per year [7]. This accretionary flux may have persisted for billions of years since the Moon’s formation, potentially contributing 1–2 wt.% carbonaceous chondrite-like material to the bulk lunar regolith [8]. Nevertheless, carbonaceous phases have rarely been identified in returned Apollo and Chang’E samples [9,10]. Unlike Earth’s atmosphere, which decelerates incoming carbonaceous debris [11], airless bodies lack atmospheric braking, enabling carbonaceous materials to strike their surfaces at nearly unattenuated velocities. Therefore, the effects of impact-induced metamorphism on carbonaceous materials remain unclear, which limits insights into extraterrestrial carbon delivery to airless bodies.

This study investigates an unusual carbonaceous grain adhering to the ilmenite surface of a basaltic clast recovered from the Chang’e-6 (CE6) lunar regolith. The microstructure, chemical composition, isotope characteristics and organic functional groups of the grain were characterized via scanning electron microscopy (SEM), transmission electron microscopy (TEM), energy-dispersive X-ray spectroscopy (EDS), electron energy-loss spectroscopy (EELS), nano-secondary ion mass spectrometry (NanoSIMS) and photo-induced force microscope infrared spectroscopy (PiFM-IR). The results suggest possible impact-induced partial graphitization of nitrogen-bearing organic-like carbonaceous matter within the dust grain. These findings provide novel insights into the delivery, chemical evolution and preservation of extraterrestrial carbon on airless body surfaces.

## RESULTS

### Unusual carbonaceous grain on a CE-6 basaltic clast surface

The CE-6 basaltic clast has dimensions of ~270 μm × 180 μm and is dominated by pyroxene and ilmenite (Figs 1a, b, S1 and Table S1). The ilmenite surface contains two rounded microcraters (RMCs), ranging from hundreds of nanometers to several micrometers in diameter, and an irregular microcrater (IMC) tens of micrometers across with abundant splash melts (Figs 1c–e, S2 and S3). An elongated grain (9 μm × 3 μm) is located at the edge of IMC (Fig. 1c). In secondary electron (SE) images, the grain is significantly brighter than the adjacent ilmenite and impact splash melts but exhibits darker contrast in backscattered electron (BSE) images (Fig. 1c–e). This indicates a relatively low average atomic number and density. Additionally, the carbon coating precludes accurate determination of grain’s intrinsic carbon abundance via EDS (See methods). Nevertheless, background-corrected EDS analyses (without subtraction of the carbon coating) indicate that the grain is more carbon-rich than the surrounding ilmenite and fragments (Figs S4, S5 and Table S2). High-magnification images further reveal that the grain consists of numerous nanoscale granules, forming a coarse, grape-like texture (Fig. 1e). This granular texture and carbon-rich composition are similar to those of chondritic porous interplanetary dust particles (CPIDPs), which are widely inferred to originate from comets [12]. Unlike canonical CPIDPs, this carbon-rich particle lacks silicate-bearing mineral assemblages (Fig. 1e), which hinders the determination of its origin. Hereafter, this unique carbon-rich dust grain from the CE-6 lunar regolith is referred to as CE6-CDP. To minimize interference from the 50 nm carbon coating on the CE6-CDP surface, a focused ion beam (FIB) foil was extracted parallel to the long axis of the grain for cross-sectional characterization (Fig. 1e; see Methods). All subsequent analyses, including TEM, EELS, NanoSIMS and PiFM-IR, were conducted on the interior of the FIB foil away from the surface carbon coating to minimize coating-derived contamination.

**Figure 1.**
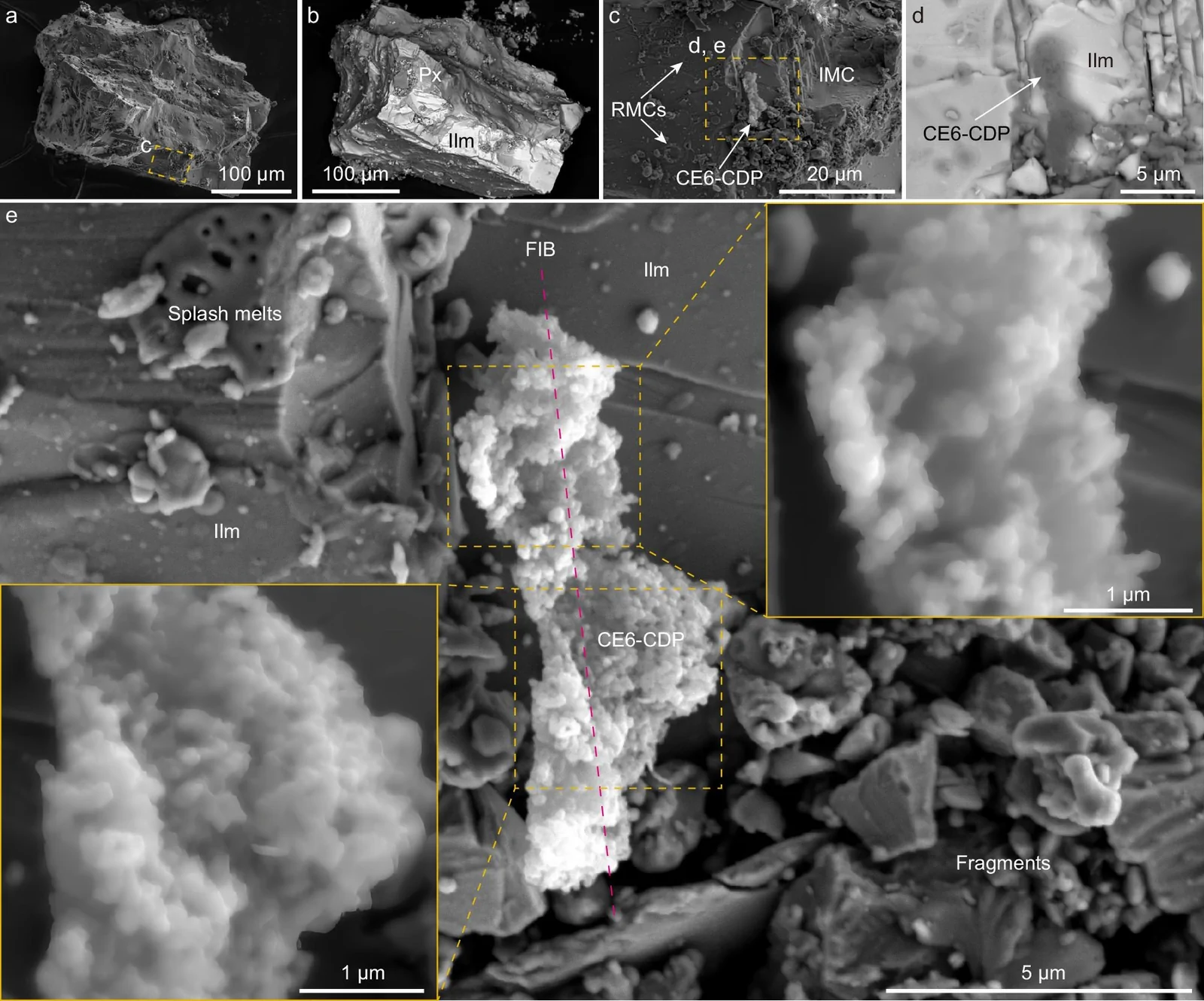
CE6-CDP adhered to the surface of a Chang’e-6 basaltic clast. (a, b) SE and BSE images of the CE-6 basaltic clast, respectively. Px: pyroxene; Ilm: ilmenite. (c) Enlarged SE image of the region outlined in a, showing CE6-CDP and several microcraters on the ilmenite surface. RMCs, rounded microcraters; IMC, irregular microcrater. (d) BSE image of CE6-CDP and the surrounding material. (e) Enlarged SE image of the region shown in c. The carmine line indicates the location of the FIB foil. The upper-right and lower-left insets represent the enlarged views of selected regions of CE6-CDP.

### Tangled and concentric poorly graphitized carbon in CE6-CDP

TEM images reveal that CE6-CDP is predominantly porous, with stalactite-like materials adhering to its bottom margin (Fig. 2a, b). EDS mapping results reveal that carbon is enriched throughout the grain, while silicon (Si) is depleted (Figs 2c and S6). This indicates the absence of silicon carbide (SiC). Unlike typical CPIDPs, which commonly contain silicate inclusions, the CE6-CDP cross section contains no silicate inclusions (Figs 2c and S6). Oxygen (O), iron (Fe) and titanium (Ti) are depleted within the porous domain of CE6-CDP but enriched along its bottom-right margin (Figs 2c, S6 and Table S3). This indicates that the stalactite-like feature is dominated by Fe–Ti oxides, consistent with the composition of lunar ilmenite. Several domains exceeding a few hundred nanometers on the right side of the CE6-CDP cross section exhibit significantly higher C enrichment than the surrounding regions (Figs 2c and S6). TEM images of these carbon-rich regions reveal tangled and concentric structures with approximately circular or polygonal morphologies (Figs 2d–f and S6). These structures are readily distinguishable from the amorphous surface carbon coating (Fig. S2). However, owing to their proximity to the carbon coating, the surface carbon-rich regions could not be reliably distinguished. Therefore, only the interior carbon-rich regions were analyzed. Additionally, the tangled and concentric textures persist across multiple TEM magnifications, indicating that these textures are intrinsic features of carbon-rich regions rather than analytical artifacts (Fig. 2b, d and e). High-resolution TEM (HRTEM) images further reveal lattice fringe spacings of ~0.340–0.342 nm within the interior carbon-rich regions, corresponding to the graphite (002) plane (Figs 2e and S7). Similar lattice fringes have been reported in numerous meteorites [13–15]. EELS spectra provide additional evidence for graphitic carbon. In the low-loss region, a γ peak occurs at ~6 eV. The core-loss region exhibits a pre-edge feature at ~285 eV, characteristic of carbon 1s → π transitions, and a distinct σ peak at ~293 eV (Fig. 2g, h) [16,17]. The TEM, EDS, HRTEM and EELS results suggest that these C-rich regions consist of graphitic carbon. In contrast, HRTEM images reveal that the Fe–Ti oxides at the CE6-CDP margin are amorphous, unlike the underlying crystalline ilmenite, consistent with impact-melted lunar ilmenite (Figs 2f, S2, S3 and S6; Table S3). The EELS spectra of the Fe–Ti oxides exhibit characteristic energy-loss peaks at the Ti *L_3_* (458.4 eV), Ti *L_2_* (462.5 eV), O K-edge (530.2 eV), Fe *L_3_* (707.5 eV) and Fe *L_2_* (720.5 eV) edges (Figs 2i and S8).

**Figure 2.**
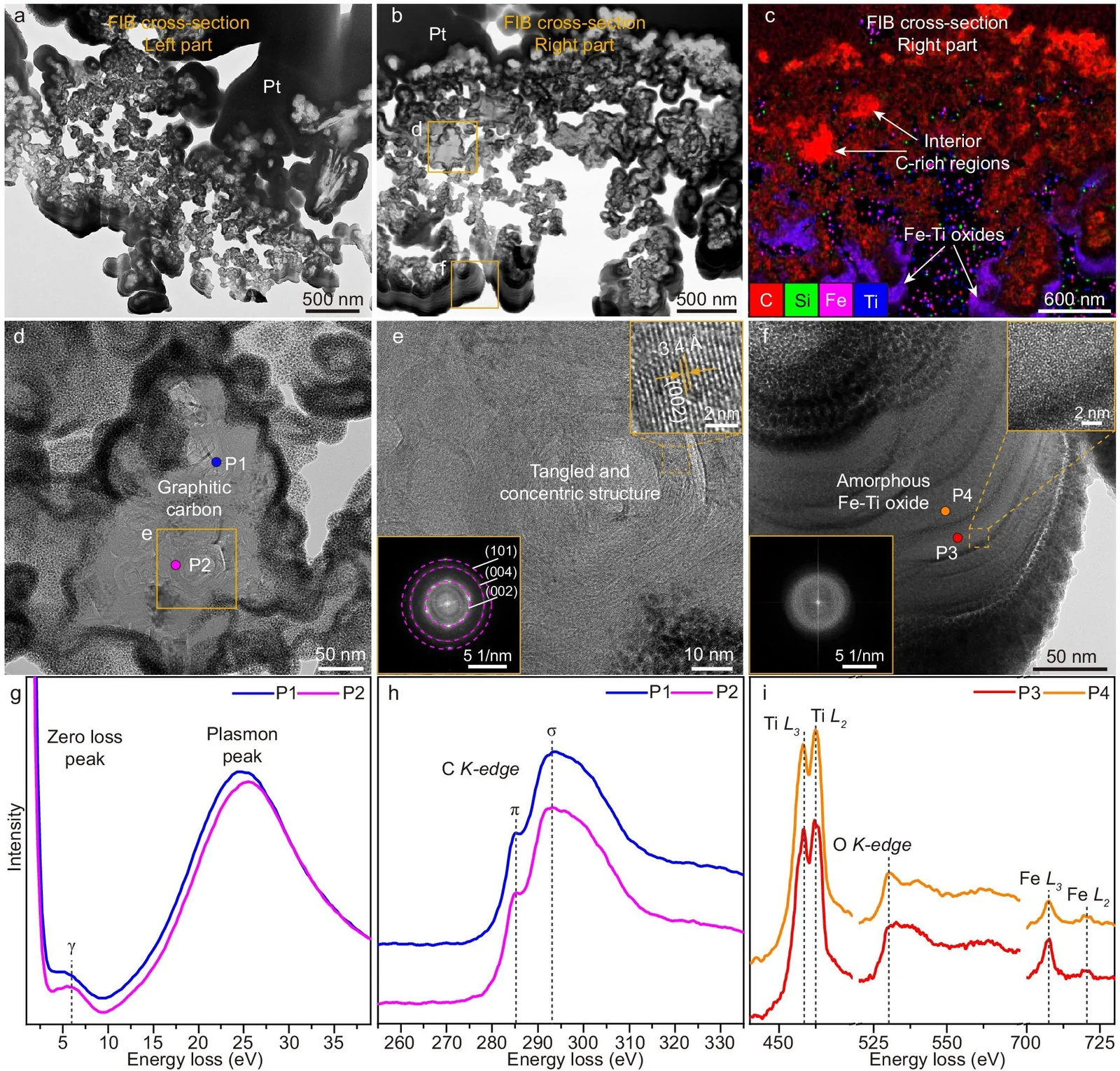
Microstructure and chemical composition of CE6-CDP. (a, b) TEM images of the left and right cross sections of CE6-CDP. (c) EDS elemental maps of the right cross section of CE6-CDP. (d) Enlarged TEM image of the region outlined in b and c, showing graphitic carbon with tangled and concentric structures. (e) HRTEM images of graphitic carbon from the selected region in d. The lower-left inset shows the corresponding fast Fourier transform (FFT) image, while the upper-right inset shows an enlarged view of the graphitic lattice fringes. (f) Enlarged TEM image of the region outlined in b and c, showing stalactitic Fe–Ti oxides with an amorphous structure. The upper-right inset shows an HRTEM image of the selected region, and the lower-left inset shows the corresponding FFT image. (g, h) Low-loss and core-loss EELS spectra of the graphitic carbon, respectively. The corresponding analysis locations are marked in d. (i) Core-loss EELS spectrum of the amorphous Fe–Ti oxides. The corresponding analysis location is marked in f. The EELS analysis locations were sufficiently separated to minimize beam-induced damage and interference between adjacent analyses. All spectra were background-subtracted and normalized (see Methods).

### Nitrogen-bearing organic-like carbonaceous material in CE6-CDP

After TEM and EELS analyses, the CE6-CDP cross section was mounted onto an indium (In) target and sputter-coated with gold (Au) for subsequent NanoSIMS and PiFM-IR measurements (Figs 3a, S9 and S10). Elemental mapping indicates that the grain is strongly enriched in both ^12^C and ^13^C (Fig. 3b, c). In contrast, the distribution of ^14^N is negatively correlated with graphitic carbon domains (Fig. 3d). The left side of the CE6-CDP cross section contains abundant ^14^N, with no significant graphitic carbon signal detected (Fig. 3d–h). The right part of the CE6-CDP cross section exhibits pronounced nitrogen depletion and graphitic carbon (Fig. 3d, i–k). Moreover, ^16^O signals remain weak throughout the grain, with two localized enrichment zones corresponding to the amorphous Fe–Ti oxide rim and lunar ilmenite. This distribution is consistent with the EDS mapping results (Fig. 3e). Virtually no carbon or nitrogen signals are detected from the Au-coated In target (Fig. 3b–e). Although the platinum (Pt) protective layer and conductive carbon coating on ilmenite exhibit weak C and N signals, their intensities are substantially lower than those detected in the CE6-CDP cross section (Fig. 3b–e). Therefore, contamination introduced during sample preparation and analysis is unlikely to dominate the signals detected within the analyzed interior CE6-CDP domains. The CE6-CDP yields ^12^C/^13^C ratios ranging from 95.00 to 99.69 (*n* = 32), corresponding to a mean δ^13^C_VPDB_ value of −25.92 ± 3.62‰ (*n* = 32) (Fig. 3l and Table S4). This isotopic signature is distinct from that of the coated carbon rod prepared using similar materials and deposition parameters (δ^13^C_VPDB_ = −2.83 ± 2.72‰; *n* = 22). The interior graphitic carbon also yields a δ^13^C_VPDB_ value of ~ −27.31‰ (*n* = 1), consistent with the bulk average δ^13^C_VPDB_ value of CE6-CDP. Across all 32 regions of interest (ROIs), including the graphitic carbon domains, δ^13^C_VPDB_ values range from −46.68‰ to 0.35‰. Notably, only one ROI exhibits a positive δ^13^C_VPDB_ value (+0.35‰) (Fig. 3l and Table S4). The carbon isotopic composition of CE6-CDP partially overlaps with that of organic matter reported in both extraterrestrial and terrestrial materials (Fig. 3m) [18–26].

**Figure 3.**
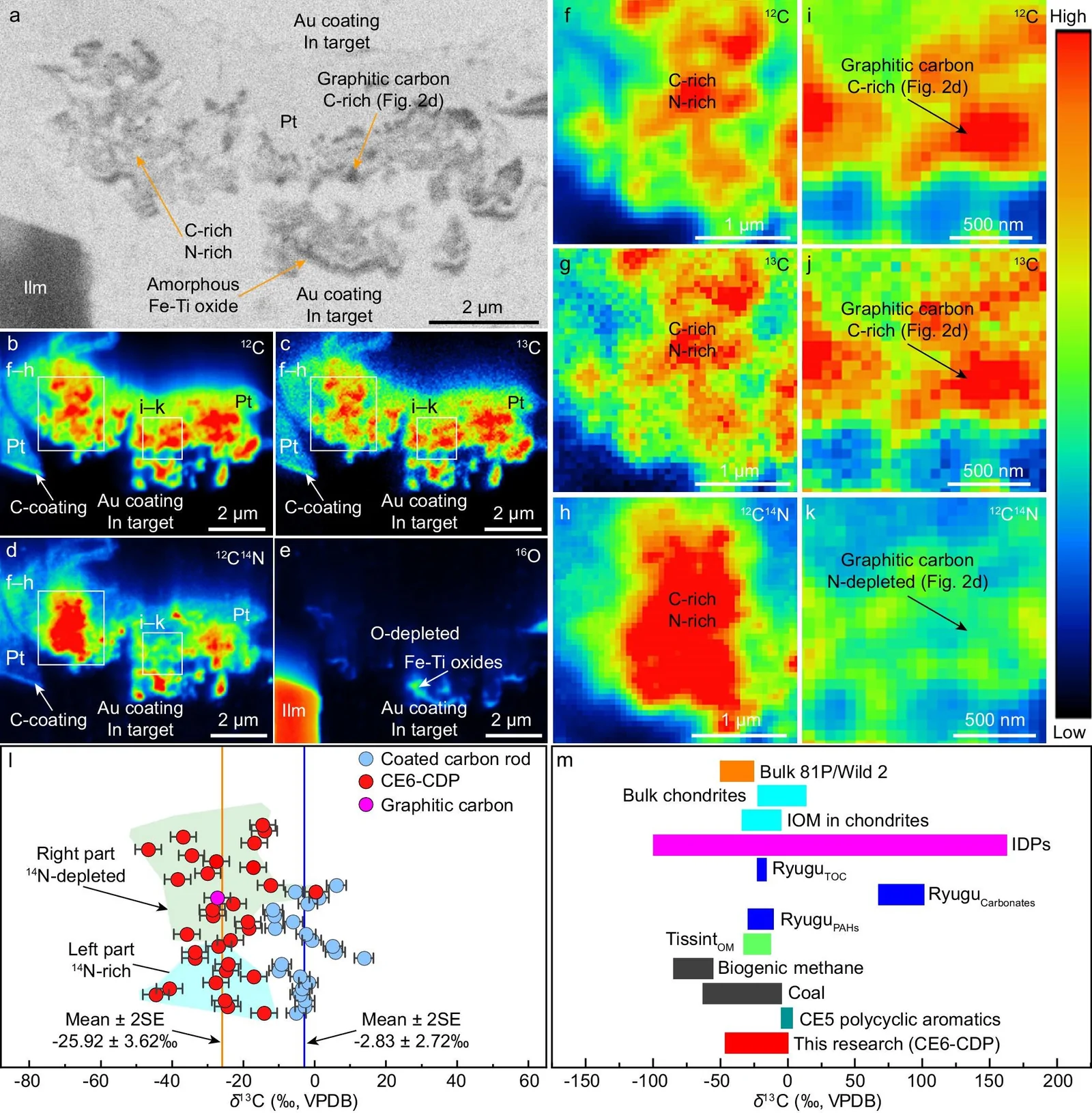
NanoSIMS analysis of the CE6-CDP cross section. (a) BSE image of CE6-CDP embedded in a custom indium (In) substrate and coated with gold (Au). (b–e) NanoSIMS isotope maps of ^12^C, ^13^C, ^12^C^14^N, and ^16^O, respectively. (f–h) and (i–k) Enlarged views of two selected regions showing the carbon–nitrogen distributions. (l) Comparison of the δ^13^C_VPDB_ values of the coated carbon rod, CE6-CDP, and the graphitic carbon in CE6-CDP. The δ^13^C_VPDB_ values of the carbon coating could not be measured from b to e owing to the limited spatial resolution. Instead, the δ^13^C_VPDB_ values were measured using identical analytical procedures. (m) Comparison of the carbon isotopic compositions of CE6-CDP, coal, and representative extraterrestrial materials. The δ^13^C values include the bulk 81P/Wild 2 sample [18], bulk chondrites and insoluble organic matter [19], IDPs [20], total organic carbon in the Ryugu sample [21], carbonates in the Ryugu sample [22], polycyclic aromatic hydrocarbons in the Ryugu sample [23], organic matter in the Tissint Martin meteorite [24], biogenic methane [25], coal (Table S4 and Ref. [25]) and CE5 polycyclic aromatic hydrocarbons [26].

The PiFM-IR spectra of the CE6-CDP cross section exhibit multiple absorption bands at ~3281 cm^−1^ (N–H stretching), ~3014 cm^−1^ (C–H stretching), ~2890 cm^−1^ (C–H stretching), ~1571 cm^−1^ (C=C/C=N stretching), ~1256 cm^−1^ (C–N stretching/N–H bending) and 994 cm^−1^ (Si–O/C–H vibrations). No distinct absorption band attributable to C=O stretching is observed near 1700 cm^−1^ (Fig. S11). These spectral features are similar to the organic signatures previously reported in carbonaceous chondrites and asteroidal meteorites [27–30]. Furthermore, the infrared spectrum of CE6-CDP is readily distinguishable from those of ilmenite (Ilm), platinum (Pt), Au coating, the In target, conductive carbon tape, and the surface carbon coating. This indicates that the organic-like carbonaceous material in CE6-CDP is unlikely to be affected by these potential contaminants (Fig. S11). This interpretation is consistent with the NanoSIMS results (Fig. 3b–e). The elemental distributions, carbon isotopic compositions, and infrared spectra are consistent with the presence of nitrogen-bearing organic-like carbonaceous material in CE6-CDP.

## DISCUSSION

### Impact-induced graphitization of carbonaceous material

Graphitic carbonaceous phases have been widely reported in diverse meteorite types [31]. However, poorly graphitized carbon has rarely been identified in Earth-collected IDPs since its first discovery nearly 40 years ago [12,32]. Moreover, although organic-like carbonaceous material and graphitic carbon have been separately reported in Apollo and Chang’e lunar samples [33–35], their coexistence within a single lunar regolith sample has not been documented. This unique occurrence requires a systematic discussion of the origin and relationship between the concentric graphitic carbon and nitrogen-bearing organic-like carbonaceous matter in CE6-CDP.

Although a ~50 nm-thick conductive carbon coating was applied during sample preparation (Methods; Fig. S2), its effect on the microanalytical results is minimal. First, CE6-CDP exhibits a distinctive porous, granular morphology distinct from the dense, layered artificial carbon coating (Figs 1e and S2). Second, the FIB cross section extends several micrometers beneath the CE6-CDP surface. The interior graphitic carbon domains are hundreds of nanometers in size, which substantially exceeds the thickness of the 50 nm coating layer (Figs 2a, b and S2). Third, HRTEM observations indicate that the conductive carbon coating is fully amorphous. Both surface and interior C-rich regions of CE6-CDP exhibit characteristic tangled and concentric graphitic textures (Figs 2c–e, and S6). Fourth, the distinct δ^13^C_VPDB_ values indicate that CE6-CDP is enriched in ^12^C relative to the artificial carbon coating (Fig. 3l). Finally, the amorphous Fe–Ti oxides adhering to the lower margin of CE6-CDP could not have formed during the carbon coating process (Figs 2 and 3). These observations suggest that the conductive carbon coating is unlikely to significantly influence the characterization of CE6-CDP and its graphitic carbon.

Previous studies have reported endogenous graphitic carbon in the lunar interior. The oxidation of this graphitic carbon has been identified as a trigger for lunar pyroclastic eruptions [8]. However, the paragenetic association of nitrogen-bearing organic-like carbonaceous matter and graphitic carbon in CE6-CDP is inconsistent with a lunar origin for the graphitic carbon. Instead, the coexistence of these two carbon phases supports an exogenous, non-lunar origin. Moreover, presolar graphite identified in Ryugu samples and numerous chondritic meteorites typically exhibit extreme carbon isotopic anomalies, with ^13^C_VPDB_ values ranging from hundreds to thousands of ‰ [18,19,24,28,36]. Therefore, the moderate δ^13^C_VPDB_ value of the concentric graphitic carbon suggests that it is unlikely to be of presolar origin.

Shock compression and thermal heating experiments have shown that organic matter is highly sensitive to variations in temperature and pressure [37,38]. Notably, concentric graphite textures synthesized from shock-treated acetylene polymers support the impact-induced metamorphism of organic precursors [39]. In CE6-CDP, the presence of adhered amorphous Fe–Ti oxides indicates that impact temperatures exceeded the melting point of lunar ilmenite (>1050°C) [40]. Moreover, the δ^13^C_VPDB_ values of the graphitic carbon are consistent with both the mean carbon isotopic composition of CE6-CDP and the δ^13^C_VPDB_ values of organic matter in terrestrial and extraterrestrial materials [8,18–26]. This indicates a common origin for the graphitic carbon and the nitrogen-bearing organic-like carbonaceous material in CE6-CDP. Additionally, the inverse spatial correlation between graphitic carbon and ^14^N distributions suggests that nitrogen was possibly partially released during the impact-induced graphitization of nitrogen-bearing organic-like carbonaceous precursors. Accordingly, impact-induced partial graphitization of exogenous nitrogen-bearing organic-like carbonaceous material is likely the most plausible mechanism for graphitic carbon formation in CE6-CDP.

However, the preservation of nitrogen-bearing organic-like carbonaceous material in CE6-CDP is inconsistent with the extreme temperatures (>1050°C) required for ilmenite melting. Furthermore, cometary sample analyses from 67P/CG indicate that porous, fragile carbonaceous grains such as CE6-CDP are unlikely to retain their original morphology during direct hypervelocity impact onto the lunar surface [41–43]. Additionally, the graphite (002) lattice fringe spacing in CE6-CDP indicates a graphitization temperature of 327–481°C, based on empirical calibrations from poorly graphitized carbon in metamorphic rocks, chondrites, and CPIDPs (Fig. S12) [44]. This temperature range is significantly lower than the melting temperature of lunar ilmenite under impact heating (>1050°C) [40]. These contrasting thermal constraints suggest that CE6-CDP is unlikely to have formed through a direct hypervelocity impact onto the lunar surface. Instead, the results indicate a plausible scenario in which CE6-CDP was ejected during an impact involving a larger carbon-rich projectile on the lunar surface. The impactor may be a C-type asteroid, a comet, a cluster of IDPs, or a Rosetta-detected comet-like fragment [43], although its precise origin remains uncertain. During this heterogeneous shock-thermal event, differential heating likely induced partial graphitization of the nitrogen-bearing organic-like carbonaceous matter and resulted in the uneven distribution of molten Fe–Ti oxides within CE6-CDP (Fig. 4).

**Figure 4.**
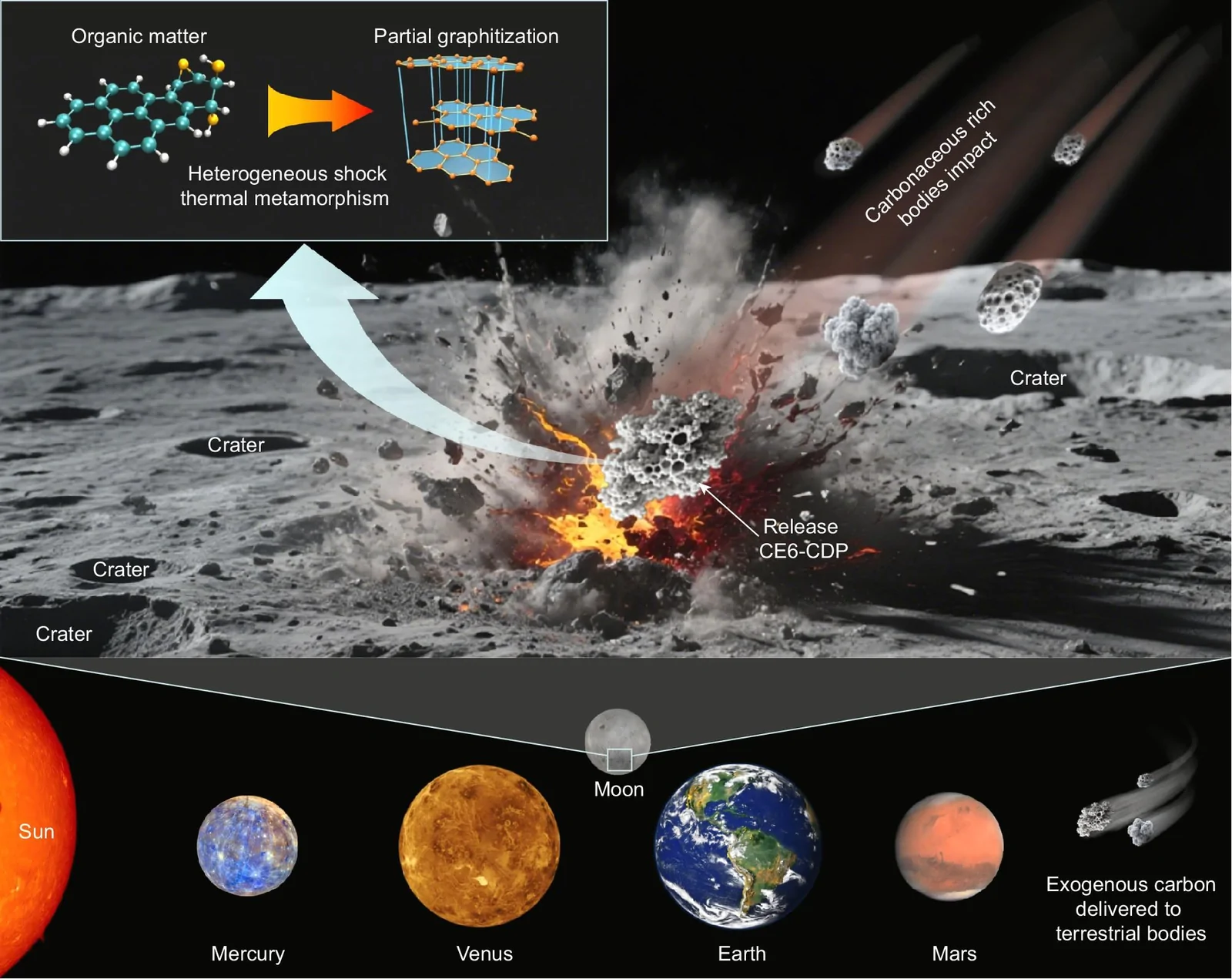
Schematic of the CE6-CDP impact on the Moon and its implications for exogenous carbon delivery to rocky planetary bodies. The sizes of all illustrated objects are not to scale.

### Implications for exogenous carbon delivery to rocky planetary bodies

The carbon-rich CE6-CDP discovered on the airless Moon and the previously reported extraterrestrial carbonaceous materials provide new insights into the role of exogenous carbon delivery to rocky planetary bodies. Notably, the Kaguya spacecraft detected a global carbon ion flux of 5.0 × 10^4^ ions/cm^2^/s across the lunar surface. This value exceeds the estimated present-day contributions from the solar wind (2.7 × 10^4^ ions/cm^2^/s) and micrometeoroids (1.5 × 10^4^ atoms/cm^2^/s). This indicates the potential existence of a widespread indigenous lunar carbon reservoir [45]. These estimates assume that the micrometeoroid population comprises 79.5% ordinary chondrites and 4.2% carbonaceous chondrites, with carbon contents of 0.2 wt.% and 3.5 wt.%, respectively [45]. However, orbital observations and dynamical simulations also suggest that near-Earth micrometeoroids are likely predominantly derived from cometary sources [6,46]. These materials typically contain considerably higher carbon contents ranging from 1 to 37 wt.% [6,46]. Particularly, micrometeoroids analogous to CE6-CDP may contain substantially higher carbon contents. These compositional uncertainties in the incoming micrometeoroid population may have resulted in an underestimation of the micrometeoroid-driven carbon flux in previous Kaguya-based estimates.

Unlike Earth’s dense atmosphere, the Moon’s tenuous exosphere permits the direct delivery of exogenous carbon from asteroids, comets, and dust particles to the lunar surface. This continuous accretion over billions of years since lunar formation may have produced carbonaceous chondrite-like materials comprising 1–2 wt.% of the mature lunar regolith [8]. Previous studies have estimated a mean impact-induced vaporization flux of ~11.6 × 10^−16^ g/cm^2^/s, which may represent a major source of lunar exospheric species [7]. Assuming that the vaporized regolith contains 1–2 wt.% carbonaceous chondrite-like material and carbonaceous chondrites contain 3.5 wt.% carbon [8,45], the resulting carbon vaporization flux from the lunar surface regolith can be estimated. The calculated flux ranges from 2.04 × 10^4^ to 4.08 × 10^4^ atoms/cm^2^/s (see Methods). This estimate does not account for factors such as ionization efficiency, consistent with the assumptions adopted in previous Kaguya-based estimates of the micrometeoroid contribution. Overall, the poorly constrained micrometeoroid composition and the continuous impact vaporization of accreted exogenous carbon may substantially contribute to the carbon ion flux detected by Kaguya. Consequently, the need to invoke a predominantly indigenous lunar carbon source may be reduced.

Nevertheless, the presence of graphitic carbon suggests that hypervelocity impacts can modify the primary properties of organic-rich carbonaceous dust particles on the lunar surface [47]. Additionally, the harsh space weathering environment of the Moon is unlikely to preserve labile organic compounds such as amino acids over geological timescales [26]. This may account for the rarity of organic signatures in lunar regolith samples [33], indicating that exogenous carbon accreted on the Moon undergoes continuous post-depositional modification. These alteration processes are likely to be more intense on Mercury. Mercury, the closest planet to the Sun, exhibits higher impact fluxes and velocities of carbon-rich micrometeorites than the Moon owing to the stronger gravitational influence of the Sun [46,48]. Therefore, exogenous carbon delivered to Mercury’s surface is more likely to be destroyed during impact and transformed into various carbon phases. The dark surface of Mercury is generally attributed to primary carbon phases formed during planetary differentiation [49,50]. However, the impact-induced graphitization of carbonaceous material in CE6-CDP, hypervelocity impact experiments, and Dawn spacecraft observations [46,51] suggest that exogenous carbon may also contribute to the dark surface features of airless bodies.

In contrast to airless bodies, extraterrestrial materials undergo a significantly different atmospheric entry process on Earth. Studies of terrestrial interplanetary dust collections have indicated that ~10 μm-sized dust grains can be efficiently decelerated in Earth’s atmosphere. Consequently, these particles undergo minimal thermal heating and are gently deposited onto Earth’s surface [52]. This process may account for the scarcity of graphitic carbon in Earth-accreted dust particles [12]. Notably, the coexistence of graphitic carbon, amorphous Fe–Ti oxides, and nitrogen-bearing organic-like carbonaceous material in CE6-CDP suggests that some extraterrestrial organic matter can survive heterogeneous shock-thermal metamorphism. This interpretation is consistent with observations of carbonaceous chondrites. In these meteorites, intense impact heating may alter surface organic layers and preserve abundant organic matter in their interiors [53]. The contrasting preservation of exogenous carbon in lunar CE6-CDP and Earth’s stratospheric dust particles highlights the key role of atmospheric conditions in preserving extraterrestrial organic materials [4]. Mars accretes ~2–3 tons of dust particles daily. The thin atmosphere and low gravity of Mars enable dust grains up to ~700 μm to survive atmospheric entry [54,55]. If present-day Mars lacks favorable conditions for endogenous organic synthesis, exogenous carbon delivered by dust may constitute a major surface organic reservoir. In contrast, Venus has a dense atmosphere and orbits significantly closer to the Sun [56]. Consequently, most incoming dust particles, including grains comparable in size to CE6-CDP, are unlikely to survive the extreme heating during atmospheric entry.

Overall, CE6-CDP represents an exceptionally rare lunar dust particle containing both nitrogen-bearing organic-like carbonaceous material and graphitic carbon (Fig. 4). Planetary conditions, including atmospheric presence, heliocentric distance and gravitational strength, may govern the evolution and fate of accreted exogenous carbon. Consequently, distinct carbon preservation pathways may operate across different solar system bodies, which requires further research.

## MATERIALS AND METHODS

The CE6 lunar soil sample (CE6C0300YJFM001) used in this study was provided by the China National Space Administration (CNSA). Sample preparation, carbon coating, and SEM–EDS analyses were performed at the Institute of Geochemistry, Chinese Academy of Sciences (IGCAS). Ultrathin foils for high-resolution microscopic characterization were prepared using FIB milling. The foils were further characterized via TEM at the Suzhou Institute of Nano-Tech and Nano-Bionics, CAS, and EELS at the Shanghai Institute of Ceramics, CAS. Carbon isotope compositions were determined via NanoSIMS at the Institute of Geology and Geophysics, CAS, using reference carbon samples analyzed under identical experimental conditions. Additionally, PiFM-IR measurements were conducted at IGCAS. The carbon atom vaporization flux from the lunar regolith was estimated using previously reported parameters. Detailed experimental procedures and calculation methods are provided in the Supplementary Materials.

## Supplementary Material

nwag573_Supplemental_File
