# Supplementary material for: Graphitization of nitrogen-bearing organic-like carbonaceous material in a dust particle from Chang’e-6 lunar samples
Source: Natl Sci Rev. 2026 Sep 4;13(19):nwag573. doi: 10.1093/nsr/nwag573 (PMC13752504; doi:10.1093/nsr/nwag573)
Supplement: Supplementary file 1 [file nwag573_supplemental_file.docx]

**Supplementary materials for “Graphitization of nitrogen-bearing organic-like carbonaceous material in a dust particle from Chang’e-6 lunar samples”**

**Authors**

Tian Zhang^1, 2^, Hong Tang^1, 2, 3*^, Xiongyao Li^1, 2, 3^, Jialong Hao^4*^, Bing Mo^1, 3^, Yuanyun Wen^1^, Futing Yin^1, 2^, Liming wan^1, 2^, Chuanjiao Zhou^1^, Wen Yu^1, 3^, Haiyan Long^1, 2^, Jianzhong Liu^1, 2, 3^

**Affiliations**

1 Center for Lunar and Planetary Sciences, Institute of Geochemistry, Chinese Academy of Sciences, Guiyang 550081, China.

2 College of Earth and Planetary Sciences, University of Chinese Academy of Sciences, Beijing 100049, China.

3 CAS Center for Excellence in Comparative Planetology, Hefei 230026, China.

4 Key Laboratory of the Earth and Planetary Physics, Institute of Geology and Geophysics, Chinese Academy of Sciences, Beijing, China.

* Corresponding author

E-mail address: [tanghong@vip.gyig.ac.cn](mailto:tanghong@vip.gyig.ac.cn); [sean_hao@mail.iggcas.ac.cn](mailto:sean_hao@mail.iggcas.ac.cn);

**Fig. S1.** EDS elemental spectra collected at two analytical spots (P1 and P2) on the CE-6 basalt clast. The inset SEM micrograph marks the positions of P1 (ilmenite) and P2 (pyroxene); the scale bar is 100 μm. Full compositional data for both spots are listed in **Table S1**. Mineral abbreviations (used throughout all following figures and tables): Ilm = ilmenite, Px = pyroxene.

**Table S1.** Chemical composition of the CE-6 basalt clast determined by EDS (data corrected for conductive carbon coating)

| Points/Elements | | O | Mg | Al | Si | Ca | Ti | Fe | Total | Notes |
| --- | --- | --- | --- | --- | --- | --- | --- | --- | --- | --- |
| P1 | Weight % | 36.5 | — | — | 0.2 | — | 29.7 | 33.6 | 100.0% | Ilm |
|  | Atomic % | 65 | — | — | 0.2 | — | 17.6 | 17.1 | 99.9% |  |
| P2 | Weight % | 33.8 | 2.2 | 1.1 | 23.8 | 6.8 | 0.6 | 31.8 | 100.1% | Px |
|  | Atomic % | 55.0 | 2.3 | 1.1 | 22.0 | 4.4 | 0.3 | 14.8 | 99.9% |  |

**Fig. S2.** TEM cross-sections of two rounded microcraters (RMCs) on ilmenite grains from the CE6 basalt clast. Panels (**a**) and (**b**) show full cross-sectional overviews of the two microcraters capped by platinum (Pt) protective layer, which overlies a ~50 nm amorphous carbon film (as shown by the FFT pattern); the lower-left insets display the top-view SEM morphology of each crater prior to FIB sectioning. Panels (**c**) and (**d**) are magnified TEM close-ups revealing subsurface vesicles and slip bands beneath the crater surfaces. Panels (**e**) and (**f**) present HRTEM micrographs of slip-bearing domains selected from panels (**c**) and (**d**), with FFT diffraction patterns (lower-left) and magnified lattice fringe insets (upper-right) provided for mineral structural identification.

**Fig. S3.** EDS elemental distribution maps of microcraters on ilmenite grain surfaces. Panels (**a, f**) are HAADF images of two microcraters at different magnifications; panels (**b-e**) and (**g-j**) display corresponding O, Si, Fe, Ti elemental maps for panels (**a**) and (**f**), respectively. Vesicles and marginal Si-rich deposits are visible within these cross-sections.

**Fig. S4.** EDS elemental spectra acquired at six analytical spots (P1–P6) across CE6-CDP and adjacent materials. The inset SEM micrograph marks the positions of all detection points, and the magnified panel (0–1 keV) highlights distinct carbon peak intensities. The spectra demonstrate prominent carbon enrichment in CE6-CDP compared with surrounding ilmenite and fragments; complete quantitative compositions for all spots are listed in **Table S2**.

**Table S2.** Compositional data of CE6-CDP and surrounding materials determined by EDS^*^

| Atomic % | P1 | P2 | P3 | P4 | P5 | P6 |
| --- | --- | --- | --- | --- | --- | --- |
| C | 40.9 | 41.1 | 53.5 | 19.9 | 23.9 | 24.9 |
| O | 36.1 | 35.8 | 29.7 | 42.4 | 21.0 | 45.8 |
| Na | — | — | — | — | — | 1.1 |
| Mg | 0.2 | 0.2 | 0.4 | — | — | 0.7 |
| Al | 0.5 | 0.4 | 1.3 | 0.1 | 0.1 | 7.2 |
| Si | 1.5 | 1.4 | 2.9 | 0.5 | 1.8 | 10.7 |
| S | — | — | — | — | — | 0.8 |
| Ca | 0.3 | 0.2 | 0.6 | 0.2 | 0.9 | 3.3 |
| Ti | 10.5 | 10.7 | 6.0 | 18.5 | 26.4 | 1.7 |
| Fe | 9.9 | 10.2 | 5.6 | 18.4 | 25.9 | 2.9 |
| Ni | — | — | — | — | — | 0.9 |
| Total | 99.9 | 100 | 100 | 100 | 100 | 99.9 |
| Position | CE6-CDP | CE6-CDP | CE6-CDP | Ilm | Ilm | Fragment |

^*^ The quantitative compositional results for all analytical spots have been background-corrected, while the signals from the conductive carbon coating were not subtracted. Accordingly, the intrinsic carbon abundance of CE6-CDP cannot be accurately quantified. Nevertheless, the carbon concentrations measured at CE6-CDP spots (P1, P2, P3) were still 16–28.6 atomic percentages (at.%) higher than the maximum carbon content recorded for surrounding spot (P6).

**Fig. S5.** EDS elemental distribution maps of CE6-CDP and adjacent materials from the CE-6 basalt clast. Panel (**a**) shows the overview labeling CE6-CDP, ilmenite substrate (Ilm), and fragments; all panels share a common scale bar of 2 μm. Panels (**b–h**) sequentially display elemental distributions of C, Si, O, Mg, Ca, Fe, and Ti. The elemental maps visually confirm strong carbon enrichment exclusively within the CE6-CDP domain, compared with the surrounding ilmenite and fragments.

**Fig. S6.** HAADF images and EDS elemental maps for CE6-CDP. Panels (**a**-**f**) show the HAADF micrograph (**a**) and corresponding elemental maps (**b–f**) of the left part of CE6-CDP; Panels (**g-l**) display the HAADF image (**g**) and elemental maps (**h–l**) from the right part. The elemental maps reveal that the entire CE6-CDP is carbon-rich and depleted in Si, O, Fe, and Ti, with the last three elements concentrated along the lower margin. The right part of CE6-CDP contains domains with anomalous carbon enrichment relative to surrounding regions. Panel (**g**) marks multiple selected areas for quantitative compositional testing, with full analytical data listed in **Table S3**.

**Table S3.** EDS-determined compositional data for selected analytical areas shown in **Fig. S6**

| Points/Elements | | C | O | Si | Ti | Fe | Total | Note |
| --- | --- | --- | --- | --- | --- | --- | --- | --- |
| Area (A1) | Atomic % | 99.63 | 0.17 | 0.17 | 0.02 | 0.02 | 100.01% | Graphitic carbon |
| Area (A2) | Atomic % | 5.47 | 53.59 | 0.85 | 22.62 | 17.47 | 100.00% | Fe-Ti Oxides |

**Fig. S7.** HRTEM characterizations of graphitic carbon hosted in CE6-CDP. Low-magnification TEM images in panels (**a**) and (**c**) show the locations of two high-resolution observation regions: surficial graphitic carbon (panel **a**) and interior graphitic carbon (panel **c**). Both domains display tangled, concentric carbon layer structures with interlayer spacings of ~3.40 Å and ~3.42 Å, as shown in the magnified lattice insets. Panels (**b**) and (**d**) are the corresponding FFT diffraction patterns of panels (**a**) and (**c**), respectively, with characteristic (002) and (101) diffraction rings of graphitic carbon clearly identified.

**Fig. S8.** EELS spectrum acquired from the ilmenite grain substrate, which serves as an internal reference standard. The inset cross-sectional TEM micrograph (upper right) shows the layered structure of the platinum (Pt) protective coating, the surface carbon film, and the underlying ilmenite substrate (Ilm); the scale bar is 100 nm. The blue dot marks the exact position of EELS signal acquisition. The plotted EELS spectrum has undergone background subtraction and normalization (see **Methods**).

**Fig. S9.** After TEM characterization, the FIB cross-sectional foil containing CE6-CDP was pressed onto a custom-made indium (In) target and coated with a gold (Au) conductive layer for further analysis.

**Fig. S10.** Schematic diagrams show the relative position of the CE6-CDP and surrounding materials in the FIB-prepared cross section; all illustrations are not to scale. Panel (**a**) displays the original cross-sectional layered structure for TEM characterization, consisting of the Platinum (Pt) protective layer, the surface carbon coating, CE6-CDP with embedded graphitic carbon domains, and the ilmenite (Ilm) substrate. Panel (**b**) presents the cross-section after it was pressed onto the custom-made indium (In) target; the ilmenite foil was fractured into several fragments during this transfer process. Panel (**c**) shows the full assembly after deposition of a thin gold (Au) conductive coating (Au) on the indium target surface. Panel (**d**) provides a three-dimensional schematic of the complete layered assembly corresponding to panel (**c**).

**Table S4.** NanoSIMS analysis results of CE6-CDP, carbon coating and coal reference

| Sample  isotope | CE6-CDP | | Carbon coating | | Coal | |
| --- | --- | --- | --- | --- | --- | --- |
|  | ^12^C/^13^C | δ^13^C_VPDB_, ‰ | ^12^C/^13^C | δ^13^C_VPDB_, ‰ | ^12^C/^13^C | δ^13^C_VPDB_, ‰ |
| ROI-1 | 96.40 | -14.15 | 95.53 | -5.14 | 101.45 | -63.27 |
| ROI-2 | 97.41 | -24.36 | 95.29 | -2.72 | 97.13 | -21.61 |
| ROI-3 | 97.48 | -25.13 | 95.29 | -2.68 | 96.12 | -11.31 |
| ROI-4 | 99.46 | -44.50 | 95.43 | -4.15 | 98.20 | -32.19 |
| ROI-5 | 99.07 | -40.71 | 95.37 | -3.52 | 93.53 | 16.06 |
| ROI-6 | 97.74 | -27.69 | 95.20 | -1.74 | 98.15 | -31.75 |
| ROI-7 | 96.69 | -17.09 | 95.43 | -4.13 | 98.06 | -30.84 |
| ROI-8 | 97.46 | -24.90 | 96.00 | -10.04 | 96.77 | -17.93 |
| ROI-9 | 97.40 | -24.29 | 95.92 | -9.23 | 96.09 | -10.93 |
| ROI-10 | 98.33 | -33.54 | 93.74 | 13.83 | 97.49 | -25.15 |
| ROI-11 | 98.33 | -33.53 | 94.49 | 5.78 | 95.45 | -4.31 |
| ROI-12 | 97.66 | -26.91 | 94.55 | 5.12 | 97.36 | -23.86 |
| ROI-13 | 97.33 | -23.62 | 95.11 | -0.80 | 96.30 | -13.13 |
| ROI-14 | 98.56 | -35.80 | 95.27 | -2.45 | 96.07 | -10.78 |
| ROI-15 | 96.82 | -18.46 | 96.10 | -11.11 | 96.20 | -12.12 |
| ROI-16 | 96.85 | -18.73 | 95.62 | -6.11 | 96.14 | -11.49 |
| ROI-17 | 97.82 | -28.49 | 96.12 | -11.33 | 97.38 | -24.10 |
| ROI-18 | 97.84 | -28.71 | 96.17 | -11.77 | 98.20 | -32.22 |
| ROI-19 | 97.26 | -22.87 | 95.22 | -1.91 | 97.64 | -26.67 |
| **ROI-20** | **97.70** | **-27.31** | 94.92 | 1.25 | 99.82 | -47.90 |
| ROI-21 | 95.00 | +0.35 | 95.56 | -5.47 | 100.60 | -55.29 |
| ROI-22 | 96.22 | -12.34 | 94.46 | 6.11 | 96.42 | -14.35 |
| ROI-23 | 98.83 | -38.43 | — | — | 98.11 | -31.38 |
| ROI-24 | 97.97 | -29.97 | — | — | 96.93 | -19.55 |
| ROI-25 | 96.70 | -17.22 | — | — | 99.05 | -40.50 |
| ROI-26 | 97.73 | -27.57 | — | — | 96.36 | -13.73 |
| ROI-27 | 98.43 | -34.52 | — | — | — | — |
| ROI-28 | 99.69 | -46.68 | — | — | — | — |
| ROI-29 | 96.67 | -16.92 | — | — | — | — |
| ROI-30 | 98.67 | -36.83 | — | — | — | — |
| ROI-31 | 96.39 | -14.09 | — | — | — | — |
| ROI-32 | 96.44 | -14.59 | — | — | — | — |
| Mean | 97.57 | -25.92±3.62 | 95.31 | -2.83±2.72 | 97.35 | -23.47 |

*****The carbon isotope features of graphitic carbon are marked by black bold numbers.

**Fig. S11.** PiFM-IR spectroscopic characterizations of the cross-sectional foil bearing CE6-CDP, together with surrounding materials, the conductive carbon tape, and the coated carbon rod. Panel (**a**) shows the photo-induced force height map (slightly tilted to show more obvious contrast); green circular markers denote six analytical spots located on CE6-CDP, ilmenite (Ilm), the platinum (Pt) protective layer, the gold conductive coating (Au) and the indium target (In), alongside a quantitative height color scale bar. Panels (**b**) and (**c**) are optical micrographs of two reference regions: conductive carbon tape and coated carbon rod, respectively. Panel (**d**) displays the full PiFM-IR spectra collected from all labeled detection spots. Characteristic vibrational peaks corresponding to N-H, C-H, C=N, C–N, N–H, Si–O stretching, as well as other organic functional groups, are marked along the wavenumber axis (cm^-1^), and the legend distinguishes the spectral curves from CE6-CDP and those from the other six distinct testing domains. In addition, the vibrations of different chemical bonds at the same peak position represent the multiplicity of spectral peak interpretation.

**Fig. S12.** Relationship between graphitization temperature and the graphite (002) lattice fringe spacing. The black solid lines indicate the uncertainty range of the mean (002) lattice fringe spacing for naturally occurring poorly graphitized carbon in terrestrial metamorphic rocks. The gray dotted line represents the overall trend. Colored symbols denote graphite identified in CI, CM, and CV chondrites and IDP CPA W7029* A. Data are from Ref. [1]. In this study, the lattice fringes of graphitic carbon in CE6-CDP correspond to a graphitization temperature of ~327–481 °C, which contradicts the melting temperature (>1050 °C) of ilmenite transitioning into adhered amorphous Fe–Ti oxides, indicating heterogeneous impact-induced heating.

**Detail description for Materials and Methods**

**Sample preparation**

The Chang’E-6 (CE6) lunar regolith sample analyzed in this study (batch ID: CE6C0300YJFM001) was provided by the China National Space Administration. Basaltic clasts were manually selected from lunar soil under a binocular microscope inside an argon‑purified inert glovebox. Isolated clasts were mounted onto conductive carbon tape and coated with a ~50 nm amorphous carbon layer for subsequent characterization. All sample preparation was completed at the Institute of Geochemistry, Chinese Academy of Sciences (IGCAS).

**SEM and EDS analysis**

A FEI Scios field‑emission scanning electron microscope (SEM) coupled with an energy‑dispersive X‑ray spectrometer (EDS) at IGCAS was utilized for morphological and compositional analysis. Secondary electron SEM images were acquired under a beam current of 0.1-0.2 nA and an accelerating voltage of 2-5 kV, while backscattered electron (BSE) images and EDS acquisitions were performed at 0.8 nA beam current and 20-30 kV accelerating voltage. Regions containing CE6-CDP were selected for high‑magnification observation. EDS point analyses and elemental mapping collected under identical instrumental conditions demonstrate that CE6-CDP exhibits stronger carbon enrichment than surrounding materials (**Fig. S4, S5, and Table S2**). Based on SEM‑EDS results, several thin foils were extracted from CE6-CDP and two microcrater sites using a focused ion beam (FIB) system, with beam currents ranging from 20 pA to 4 nA and accelerating voltages of 5-30 kV.

**TEM, EDS and EELS analysis**

These FIB foils were characterized on a Talos F200X field‑emission scanning transmission electron microscope (FE‑STEM) at the Suzhou Institute of Nano‑Tech and Nano‑Bionics, CAS (SINANO). Microstructural features were imaged via bright‑field TEM and high‑angle annular dark‑field (HAADF) STEM, and nanoscale crystal structures were resolved using high‑resolution TEM (HRTEM). EDS compositional mapping was performed on targeted regions of each FIB foil, with each mapping area acquired for more than 3 min.

Electron energy loss spectroscopy (EELS) measurements were conducted on a Hitachi HF5000 aberration‑corrected STEM operated at 200 kV, equipped with a Gatan GIF Quantum ER Model 965 parallel EELS spectrometer at the Shanghai Institute of Ceramics, CAS (SICCAS). All EELS spectra were recorded in dual EELS mode with a probe current of 100 pA. Carbon (C) edges were collected from graphitic carbon domains, whereas titanium (Ti) and iron (Fe) edges were acquired from amorphous Fe-Ti oxides and the ilmenite matrix, respectively. All point‑mode spectra covered both low‑loss and core‑loss energy windows with an energy resolution of 0.25 eV, and raw datasets were processed using Digital Micrograph software. Energy drift was calibrated against the zero‑loss peak, and the corresponding shift was applied to all spectra within each acquisition series. Background signals and plural scattering contributions were subtracted from all spectra. Sequential acquisition of Ti, Fe, and O core‑loss spectra introduced noticeable energy drift and oxidation artifacts; therefore, single‑point EELS spectra collected from the pure ilmenite substrate served as an internal reference for secondary drift correction. All spectra plotted in the manuscript and the supplementary figures were normalized to identical intensity for direct visual comparison.

**NanoSIMS measurement**

The original thin carbon coating covering the surfaces of CE6-CDP and ilmenite grains is too thin for reliable direct isotopic analysis. We therefore fabricated a rod-shaped carbon coating reference using the identical materials and deposition parameters as the original surface coating. This synthetic coated carbon rod and the FIB foils bearing CE6-CDP were co-mounted onto the two independent custom-fabricated indium (In) targets. All specimens were subsequently coated with a thin conductive gold (Au) film via sputter deposition prior to isotopic characterization. Carbon isotopic compositions of CE6-CDP and laboratory carbon standards were measured with a CAMECA NanoSIMS 50L secondary ion microprobe at the Institute of Geology and Geophysics, Chinese Academy of Sciences (IGGCAS). A mass resolution of ~8000 (M/ΔM at 10% peak height) was achieved during data collection. Secondary ions of ^12^C^-^, ^13^C^-^, ^16^O^-^ and ^12^C^14^N^-^ were simultaneously detected via electron multipliers by rastering a focused Cs^+^ primary ion beam (0.6-1 pA, 80-100 nm spot size). Mapping dimensions were set to 10 × 10 μm^2^ (256 × 256 pixels) for carbon rods and 15 × 15 μm^2^ (256 × 256 pixels) for CE6-CDP, with a uniform pixel size of 60 nm. For carbon reference rods, the dwell time per pixel was 5 ms (327 s per layer), with six layers measured in total; for CE6-CDP, the dwell time was also 5 ms per pixel (655 s per layer), across 80 stacked layers.

Carbon isotope measurements were calibrated using a coal reference material with a known carbon isotope composition of δ^13^C = −23.45‰. The coal reference was mounted and analyzed under the same instrumental conditions as the CE6-CDP and coated carbon rod samples. The measured ^13^C/^12^C ratio of the coal reference was used to determine the instrumental mass fractionation (IMF) factor, which was then applied to correct the measured ^13^C/^12^C ratios of the unknown ROIs [2]. The corrected carbon isotope compositions are reported relative to VPDB using:

δ^13^C_VPDB_ = {(^13^C/^12^C) _sample_/(^13^C/^12^C) _VPDB_-1} × 1000‰,

where the standard ^13^C/^12^C ratio of Vienna Pee Dee Belemnite (VPDB) is 0.0112372. The electron multiplier dead time was set to 44 ns, and dead-time correction was performed during image data reduction using ImageJ/OpenMIMS [3]. Quasi-simultaneous arrival correction was not applied because the measured K value, defined as the ratio of secondary ions ejected per primary ion, was very low for ^12^C⁻ in this study, approximately 0.001 [4]. Therefore, the QSA effect was considered negligible under the present analytical conditions. Prior to the isotope measurements, the peak-height distribution of the electron multipliers was evaluated by monitoring the PHDmax values. No obvious aging effect or deterioration in detector response was detected during the analytical session [5]. Regions of interest (ROIs) were defined based on the ^12^C⁻ ion images. ROIs smaller than twice the primary beam spot area were excluded to avoid unreliable isotope ratios, and carbon isotope values with uncertainties larger than twice the standard error were not considered further.

**PiFM-IR measurement**

After NanoSIMS analysis, the CE6-CDP was stored in an argon-filled glove box and then transferred by a seal box to conduct the photo-induced force microscopy-infrared spectroscopy (PiFM-IR) (Molecular Vista 75) analysis at IGCAS. Prior to infrared spectral acquisition, a topographic AFM pre-scan was performed on CE6-CDP to locate target analytical domains, with a scanning area of 18.5 × 15.7 μm^2^ and a pixel resolution of 256 × 256 pixels. Single-point PiFM-IR spectra were collected in surface/bulk-sensitive mode with an effective probe depth of ~20 nm, using an OPO/DFG dual-band laser covering spectral windows of 625–2000 cm^-1^ and 2280–4385 cm^-1^. The fixed spectral resolution was set to 1 cm^-1^, and the nominal lateral spatial resolution of the system was better than 10 nm. To avoid the thermal degradation of carbon-bearing samples during measurement, a low laser power of 0.5 vol% (volume percentage) was applied, with an integration time of 40 s for each individual spectrum.

Spectra of other phases on the FIB cross-section were acquired under identical instrumental conditions, including ilmenite substrate (Ilm), platinum protective layer (Pt), gold conductive coating (Au), and indium target (In). To avoid contaminating the probe, the optical micrographs were separately captured for conductive carbon tape and coated carbon rod, while their PiFM-IR spectra were measured with consistent acquisition parameters. All raw spectra underwent identical smoothing and uniform intensity normalization to facilitate direct comparative analysis.

**Estimate** **the vaporization flux of carbon from surficial lunar regolith**

Calculations of meteoroid‑impact‑induced vaporized carbon atomic flux (*F*_C-atoms_) rely on stepwise conversion of mass fractions via the following equation:

*F*_C-atoms_ = *F*_all_ × *f_1_* × *f*_2_ × $\frac{N_{A}}{M_{C}}$

Here, *F*_all_ refers to the current total vaporization flux of lunar regolith induced by meteoroid impacts, with a value of 11.6 × 10^-16^ g/cm^2^/s [6]; *f_1_* represents the mass fraction of carbonaceous chondrite‑like exogenous material in lunar regolith (1-2 wt.%) [7]; *f_2_* corresponds to the carbon mass fraction within carbonaceous chondrite analogs (~ 3.5 wt.%) [8]; *N_A_* is Avogadro’s constant (6.02 × 10^23^ atoms/mol) ; *M_c_* is the molar mass of carbon, with an adopted value of 12.01 g/mol. To enable direct comparison with the micrometeoritic carbon flux derived from Kaguya orbital observations (1.5 × 10^4^ atoms/cm^2^/s), all calculated results are reported in identical atomic flux units. Several simplifying assumptions were adopted in this model, and key unquantified parameters such as ionization efficiency were not incorporated.

**References**

1.	Rietmeijer FJ, Mackinnon ID. Poorly graphitized carbon as a new cosmothermometer for primitive extraterrestrial materials. *Nature*. 1985; **315**(6022): 733-736.

2.	Lin Y, El Goresy A, Hu S *et al.* NanoSIMS analysis of organic carbon from the Tissint Martian meteorite: Evidence for the past existence of subsurface organic‐bearing fluids on Mars. *Meteorit. Planet. Sci*. 2014; **49**(12): 2201-2218.

3.	Poczatek C, Kaufman Z, Lechene C. OpenMIMS ImageJ plugin guide. *Harvard Medical School (Boston, Massachusetts, USA)*. 2012: 1-18.

4.	Jones C, Fike DA, Peres P. Investigation of the quasi‐simultaneous arrival (QSA) effect on a CAMECA IMS 7f‐GEO. *Rapid Commun. Mass Spectrom*. 2017; **31**(7): 623-630.

5.	Hao J-L, Zhang L-P, Yang W *et al.* NanoSIMS sulfur isotopic analysis at 100 nm scale by imaging technique. *Front. Chem*. 2023; **11**: 1120092.

6.	Pokorný P, Janches D, Sarantos M *et al.* Meteoroids at the Moon: orbital properties, surface vaporization, and impact ejecta production. *J. Geophys. Res.-Planets*. 2019; **124**(3): 752-778.

7.	McCubbin FM, Vander Kaaden KE, Tartèse R *et al.* Magmatic volatiles (H, C, N, F, S, Cl) in the lunar mantle, crust, and regolith: Abundances, distributions, processes, and reservoirs. *Am. Miner*. 2015; **100**(8-9): 1668-1707.

8.	Yokota S, Terada K, Saito Y *et al.* KAGUYA observation of global emissions of indigenous carbon ions from the Moon. *Sci. Adv*. 2020; **6**(19): eaba1050.
